# Molecular Epidemiology of Human Papillomavirus Infection Among Chinese Women With Cervical Cytological Abnormalities

**DOI:** 10.3389/fpubh.2022.820517

**Published:** 2022-05-17

**Authors:** Eliza Lai-Yi Wong, Annie Wai-Ling Cheung, Zigui Chen, Amy Yuen-Kwan Wong, Apple Chung-Man Yeung, Peter Sen-Yung Yau, Paul Kay-Sheung Chan

**Affiliations:** ^1^Centre for Health Systems and Policy Research, The Jockey Club School of Public Health and Primary Care, Faculty of Medicine, The Chinese University of Hong Kong, Hong Kong, Hong Kong SAR, China; ^2^Department of Microbiology, Faculty of Medicine, The Chinese University of Hong Kong, Hong Kong, Hong Kong SAR, China

**Keywords:** cervical neoplasia, cervical cancer screening, HPV prevalence, HPV genotypes, cytological abnormalities

## Abstract

**Background:**

Virtually all invasive cervical cancers are caused by persistent genital human papillomavirus (HPV) infection. Therefore, HPV-based screening becomes an essential tool as one of the cervical prevention strategies to reduce the disease burden. Population-specific epidemiologic information on HPV infection among women with cytological abnormalities is essential to inform the strategy of HPV-based screening programme. The study also explored the presence of cutaneous HPV types (Beta-β and Gamma-γ) in cervical infections.

**Methods:**

A cross-sectional study on Chinese women aged ≥25 years who were referred to public specialist out-patient clinics for colposcopy or further management of cervical cytological abnormalities were recruited between 2015 and 2016 in Hong Kong. HPV was detected and typified by the novel PCR-based Next-Generation Sequencing (NGS) strategies.

**Results:**

The overall HPV infection rate was 74% and detected in 222 of the 300 respondents, with the prevalence of cutaneous HPV infection being 2.3%. The overall prevalence of HPV infection among women with current cytological abnormalities was 79.1% (197/249). The age-specific prevalence of HPV (any-type HPV infection) among women with cytological abnormalities reached the first peak with 87.9% in the age group of 35–39 years and gradually declined to 56.0% at 55–59 years. While a second peak occurred at 65 years or above (92.9%). HPV58 (13.7%), HPV52 (11.7%), HPV53 (11.2%), HPV16 (10.0%), HPV18 (5.2%), and HPV51 (5.2%) were the top five high-risk HPV genotypes among women with cytological abnormalities. Any-HPV type infection was significantly associated with an abnormal cervical smear (OR = 3.7; 95% CI 2.0–7.1), and high-risk HPV infection was also significantly associated with an abnormal cervical smear (OR = 6.3; 95% CI 3.0–13.5).

**Conclusion:**

New evidence on the second peak of HPV infection at ≥65 years old suggests the necessity to review the current guideline for the cervical screening program extending to age 65 and above. Moreover, the high prevalence of two HPV genotypes—high-risk HPV51 and potential high-risk HPV53, among women with cytological abnormalities—suggests further research work is needed to confirm the contributory role of HPV51 and HPV53 in cervical cancer and the need for inclusion in the next generation of the HPV vaccine.

## Introduction

Cervical cancer is the fourth most common cancer in women with more than 311,000 women dying from the disease in 2018, accounting for 7.5% of all female cancer deaths globally (1). Cervical cancer is caused by persistent genital human papillomavirus (HPV) infection, with its DNA sequences detected in over 99% of invasive cervical cancers. To date, more than 200 types of HPV have been recognized and categorized into five major phylogenetic genera, namely alpha- (alpha-PVs), beta- (beta-PVs), gamma- (gamma-PVs), mu- (mu-PVs), and nu- (nu-PVs) papillomavirus. While alpha-PVs infect epithelial cells in genital mucosa and all high-risk HPVs are subgroups of these genera, which is often referred to as high-risk genital HPV (Hr-HPV). Oral mucosa or skin (cutaneous epithelia) on the other hand, would be presented with all five genera of HPV types (2).

The cutaneous HPV types are mainly from beta-PVs and gamma-PVs which are shown to be highly prevalent in patients with skin lesions, such as benign skin warts and actinic keratosis (AK) that results from chronic exposure to ultraviolet radiation. Beta-PVs cooperate well with UV radiation that essentially promotes skin cancer, in particular non-melanoma skin cancers (NMSCs) (3). Thus, the relationship between cutaneous HPV types and skin cancer is well-established. In addition, there is growing evidence that beta-PVs and gamma-PVs are detected at mucosa sites (4). Findings revealed that beta-PVs were prevalent in genital skin (81.6%) followed by forearm skin (64.4%) and anal mucosa (33.3%), suggesting the possible etiological role of cutaneous HPV types in cervical infections. However, little attention has been paid to the role of cutaneous HPV types in cervical infections (5). Most studies have focused on the association between alpha-PVs and cervical cancer, as Hr-HPV plays a crucial role in the development of invasive cervical cancer. Further studies are therefore required to better understand the cutaneous HPV types in mucosotropic cancers, especially in cervical cancer.

In Hong Kong (HK), cervical cancer has always been within the top ten of the commonest cancer among females with an average of 500 new cases are diagnosed every year. The age-standardized incidence rate was 8.4 per 100,000 standards in the Hong Kong population (6). In 2017, cervical cancer ranked number nine in female cancer mortality in Hong Kong with a total of 150 women dying from this cancer, accounting for 2.6% of female cancer deaths (6). It is well recognized that persistent high-risk HPV infection is a strong predictive factor of cervical cancer—HPV detection, therefore, plays a paramount important role in cervical cancer prevention. Population-specific prevalence data on HPV infection is critical for constructing an effective HPV-based cervical screening strategy, either applied as a primary or adjunct tool. Previous local studies mainly focused on women who attended cervical cancer screening in which the majority had normal cytology results (7, 8). In this study, we focused on women referred for further management of abnormal cytology to generate results that are comparatively relevant to the consideration of the HPV-test as an adjunctive or standalone tool in cervical cancer screening among the Chinese women population. Thus, we provide an updated epidemiological profile of HPV infection after 10 years of reported data among women with different cytological statuses in the local population (9). The study also provided an opportunity to explore the presence of cutaneous HPV types (Beta-β and Gamma-γ) in cervical infections among the Chinese population in South-East Asia.

## Methods

### Study Subjects

Women aged 25 years or older who were referred to the Specialist Out-patient Clinic (SOPC) under the Hospital Authority (HA) for colposcopy or further check-ups of cervical cytological abnormalities were recruited between September 2015 and December 2016. Patients who are known to be immunocompromised such as systemic lupus erythematous, long-term steroids user, and those without intact of the cervix were excluded from the study.

### Study Design

A cross-sectional survey was conducted using a structured questionnaire to elicit information on demographic, health status, gynecological history, sexual, and lifestyle behavior. A laboratory test was performed to confirm the cervical disease and HPV infection status based on cytology smears repeated in the colposcopy clinic and ascertained by colposcopy, and biopsy was performed when clinically indicated. Histology results were applied when cytology was unavailable. This study was approved by the Research Ethics Committee (CUHK-NTEC CREC) at the first author's institution.

#### Laboratory Test for HPV Detection and Genotyping

Cervical scrape samples were used for DNA extraction (10) using the QIAamp DNA Mini Kit (11) and HPV infection was determined by HPV genotyping and sequencing. The novel PCR-based Next-Generation Sequencing (NGS) strategies were used to detect the full spectrum of HPVs including alpha-PVs, beta-PVs, and gamma-PVs, as previously described (12). Briefly, a pair of unique 12-bp barcodes were introduced to the PCR amplicon by forward and reverse primers. Successful amplicons with predicted fragment sizes were pooled at approximately equal molar DNA concentrations and sequenced on an Illumina MiSeq (13) using 150-bp paired-end reads. The demultiplexed paired-end Illumina short reads passing the quality filter (≥Q20 and ≥50-bp), were merged into single reads using FLASh v1.2.11 and blasted against genomes online database (gold) papilloma virus (PV) reference database using UPARSE software. Our PV reference database contains 387 fully characterized human (*n* = 225) and animal (*n* = 162) PV types, and 467 potential novel partial PV sequences. An operation taxonomic unit (OTU) count table giving the number of reads per sample per OTU, was created using a 95% identity threshold utilizing in-house developed scripts. The OTU taxonomy was classified at the type level based on sequence homology to the reference database: if OTUs hitting the reference database had ≥90% identities to a characterized PV type, they represented known viruses; while those with 60–89% identities were regarded as “uncharacterized” types and assigned with a unique identity. An HPV type was considered positive if the reads were ≥50. Twelve alpha-HPV types (HPV16, 18, 31, 33, 35, 39, 45, 51, 52, 56, 58, and 59) classified as “carcinogenic to humans” (Group 1) and potential high-risk types (HPV53, 66, 67, 68, 70, and 82) were considered as high-risk HPV types in this study.

### Statistical Analysis

Data were analyzed using the statistical software R version 4.0.5 and IBM SPSS version 26.0 with a dual data entry. Over 10% of the database was further audited to ensure accuracy and completeness. Descriptive statistics of respondents' characteristics according to HPV infection status and cytological abnormalities were reported. Univariate analyses including Chi-Squared or Fisher's exact test were used for categorical variables, and a *t*-test was used for continuous variables. Multivariate analysis by logistics regression was carried out to explore the association of cervical cytological abnormalities and HPV infection status, and other epidemiological factors if applicable. A *p*-value < 0.05 is considered statistically significant.

## Results

### Demographic Characteristics and Health Status

Among the 300 recruited respondents, the mean age was 46 years (SD = 11.4), ranging from 25 to 76 years (Table 1). Most respondents had completed secondary education or above (75.6%) were married or cohabiting (75.7%), and lived in Hong Kong for more than 10 years (79.3%). More than 80% were active alcohol drinkers but only a small number were ever smokers (13.3%). For their sexual and lifestyle behavior, more than half of them (55.0%) had first sexual intercourse under 21 years of age and 5.7% reported a history of the sexually transmitted disease (STD). In terms of protective behavior, only around 10% of respondents had received HPV vaccination.

**Table 1:** Demographics and health status by cervical cytological status.

	**Cervical cytological status N (Col %)^∧^**			
**Variable** **(*N*)**	**Normal** **(*N* = 51)**	**Abnormal** **(*N* = 249)**	**Total** **(*N* = 300)**	***P*** **-value**
Age Mean (S.D.) (300)	49.6 (9.6)	45.0 (11.6)	45.8 (11.4)	0.009^*^
Education (299)				0.652
Primary or below	15 (29.4)	58 (23.4)	73 (24.4)	
Secondary	28 (54.9)	150 (60.5)	178 (59.5)	
Tertiary or above	8 (15.7)	40 (16.1)	48 (16.1)	
Marital Status (300)				0.328
Single	2 (3.9)	25 (10.0)	27 (9.0)	
Married/Cohabited	42 (82.4)	185 (74.3)	227 (75.7)	
Separated/Widow	7 (13.7)	39 (15.7)	46 (15.3)	
No. of years lived in HK (300)				0.559
≤ 10	9 (17.6)	53 (21.3)	62 (20.7)	
≥11	42 (82.4)	196 (78.7)	238 (79.3)	
Smoking Status (300)				0.124
Never smokers	48 (94.1)	215 (86.4)	263 (87.7)	
Ever smokers	3 (5.9)	34 (13.7)	37 (13.3)	
Alcohol Drinking (299)				0.860
Occasional drinker	10 (19.6)	46 (18.5)	56 (18.7)	
Active drinker	41 (80.4)	202 81.5)	243 (81.3)	
Age at 1st sexual intercourse (300)				0.231
≥Aged 21	27 (52.9)	109 (36.3)	136 (45.3)	
≤ Aged 20	24 (47.1)	140 (56.2)	164 (55.0)	
No. Lifetime Sexual Partners (300)				0.283
≤ 1	24 (47.1)	97 (39.0)	121 (40.3)	
≥2	27 (52.9)	152 (61.0)	179 (59.7)	
HPV Vaccination (299)				0.820
No	46 (90.2)	221 (89.1)	267 (89.3)	
Yes	5 (9.8)	27 (10.9)	32 (10.7)	
History of sexually transmitted disease (STD) (299)				0.947
No	48 (94.1)	234 (94.4)	282 (94.3)	
Yes	3 (5.9)	14 (5.6)	17 (5.7)	
Total	51 (100)	249 (100)	300 (100)	

∧ *The value did not add up to the column total due to the missing responses and are not add up to 100% due to the rounding*.

* *p-Value < 0.05 (chi-square or Fisher exact test was used for categorical variables and t-test was used for continuous variables)*.

Cytological abnormalities were detected in 83% (249/300) of all cervical smears repeated in the colposcopy clinic. Univariate analysis revealed that persistent abnormal cytology was significantly associated with younger age (45.0 vs. 49.6 years, *p* = 0.009) (Table 1). In terms of the cervical pathology profile including histology reports with abnormalities, 191 cases (76.7%) had low-grade squamous intraepithelial lesion (LGSIL) including Inflammatory/atypia (164 cases) and cervical intraepithelial neoplasia (CIN1) (27 cases), and 58 cases (23.3%) had high-grade squamous intraepithelial lesion (HGSIL) including CIN2 (29 cases) and CIN3 (29 cases) (Table 2).

**Table 2:** HPV detection among different cytological status.

	**HPV detection**			**Type of HPV infection**	
**Cytological status**	**Negative** ***N*** **(%)**	**Positive any-type alpha-HPV *N* (%)**	**Total** ***N*** **(%)**	**Low-risk only** **alpha-HPV** ***N*** **(%)**	**High-risk^#^** **alpha-HPV *N* (%)**
Normal	26 (51.0)	25 (49.0)	51 (100)	13 (25.5)	12 (23.5)
Abnormal	52 (20.9)	197 (79.1)	249 (100)	38 (15.3)	159 (63.9)
LGSIL	49 (25.6)	142 (74.3)	191 (100)	37 (19.4)	105 (55.0)
Inflammatory/atypia	42 (25.6)	122 (74.4)	164 (100)	32 (19.5)	90 (54.9)
CIN 1	7 (25.9)	20 (74.1)	27 (100)	5 (18.5)	15 (55.6)
HGSIL	3 (5.8)	55 (94.8)	58 (100)	1 (1.7)	54 (93.1)
CIN 2	0 (0.0)	29 (100)	29 (100)	1 (3.5)	28 (96.6)
CIN 3	3 (10.3)	26(89.6)	29 (100)	0 (0.0)	26 (89.7)
Overall	78 (26.0)	222 (74.0)	300 (100)	51 (17.0)	171 (57.0)

# *High-risk HPV infection: including high-risk genotypes (16, 18, 31, 33, 35, 39, 45, 51, 52, 56, 58, and 59) and potential high-risk genotypes (53, 66, 67, 68, 70, and 82)*.

### Prevalence of HPV Infection

HPV infection was detected in 74.0% (222/300) of respondents, as shown in Table 2. The overall prevalence of mucosal HPV infection among women with cytological abnormalities was 79.1% (197/249), which was considerably higher than those with normal cytological results (49.0%, 25/51). The prevalence of any-type HPV infection among those with LGSIL (including inflammatory/atypia and CIN1) and HGSIL (including CIN2 and CIN3) was 74.3 and 94.8%, respectively. Whereas, the prevalence of Hr-HPV genotypes among respondents with LGSIL and HGSIL was 55.0 and 93.1%, respectively. Among different abnormal cervical cytological statuses, the prevalence of Hr-HPV infection was 54.9% in inflammatory/atypia, 55.6% in CIN1, 96.6% in CIN2, and 89.7% in CIN3. The overall prevalence of cutaneous HPV infection (Beta-β and Gamma-γ) was 2.3% (7/300) in which all of these respondents also had cytological abnormalities (2.8%, 7/249). Among them, 85.7% (6/7) were detected with gamma-HPV types while only one of them (14.3%) was infected with beta-HPV (Table 3). Due to the small-scale cutaneous HPV detection, the distribution of beta-/gamma-HPV infection among abnormal cytological status was not presented.

**Table 3:** The relationship between mucosal and cutaneous HPV genotypes.

**Mucosal HPV genotypes**		**Cutaneous HPV genotypes**			
**Genus α**	**Genus β**	**Genus γ**			
	**HPV105** ***N*** **= 1**	**HPV101** ***N*** **= 3**	**HPV108** ***N*** **= 1**	**HPV121** ***N*** **= 1**	**HPV147** ***N*** **= 1**
Low-risk	0	3	0	0	0
HPV42	0	1	0	0	0
HPV43	0	1	0	0	0
HPV62	0	1	0	0	0
High-risk	0	3	1	1	1
HPV16	0	0	1	0	0
HPV18	0	0	0	1	0
HPV53	0	1	0	0	0
HPV56	0	0	0	0	1
HPV58	0	1	0	0	0
HPV70	0	1	0	0	0
Overall	0	6	1	1	1

#### Type-Specific Prevalence of HPV Infection

**Figure 1:**
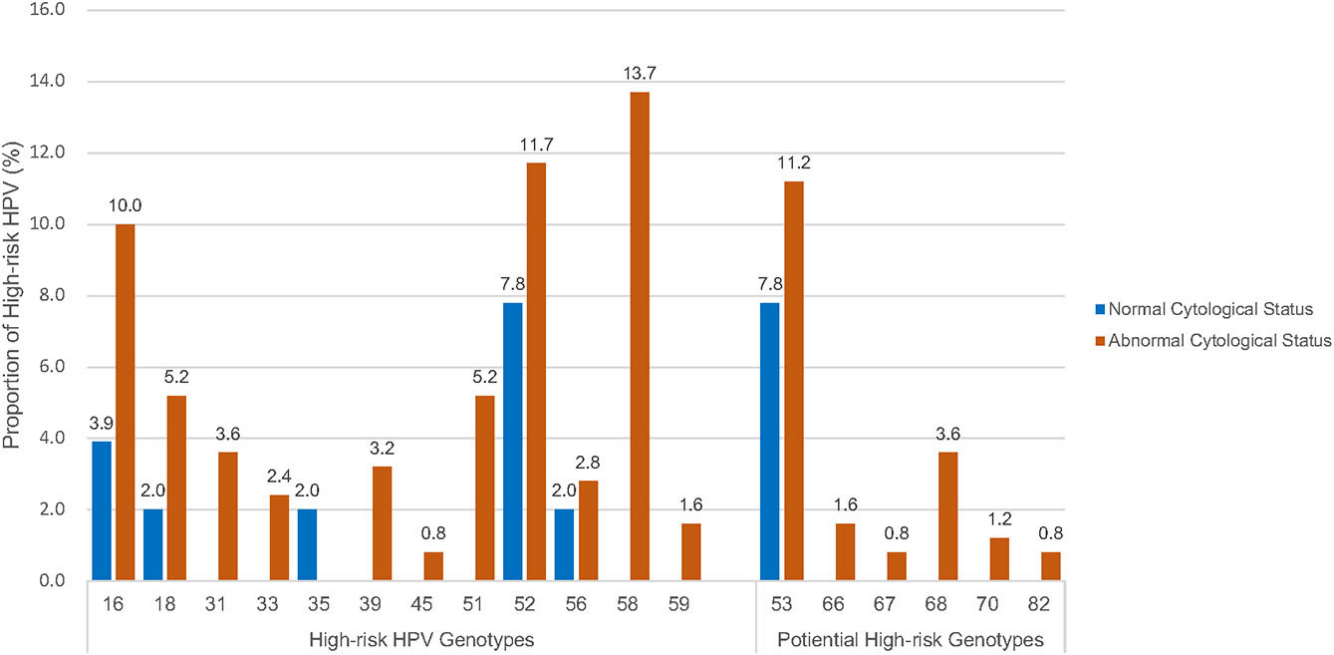
Distribution of high-risk HPV genotypes^#^ by cytological status. ^#^High-risk HPV genotypes: including high-risk genotypes (16, 18, 31, 33, 35, 39, 45, 51, 52, 56, 58 and 59) and potential high-risk HPV genotypes (53, 66, 67, 68, 70, 82).

A wide spectrum of HPV genotypes was detected in this study. A total of 38 HPV genotypes were identified, of which 18 Hr-HPVs including potential Hr-HPV genotypes were found, namely, HPV16, 18, 31, 33, 35, 39, 45, 51, 52, 53, 56, 58, 59, 66, 67, 68, 70, and 82 (Figure 1). Figure 1 reveals that HPV58 (13.7%), 52 (11.7%), 53 (11.2%), 16 (10.0%), 18 (5.2%), and 51 (5.2%) were the top five most prevalent Hr-HPV genotypes among women with cytological abnormalities. Of these, the top Hr-HPV infection types were HPV53 among those with inflammatory/atypia, HPV53, 16 and 18 among those with CIN1, HPV52 among those with CIN2, and HPV58 among those with CIN3. The remaining Hr-HPV genotypes ranged from 0.8 to 3.6%. In addition, five Hr-HPV genotypes were found among women with normal cytological status including HPV52, 53, 16, 18, 35 and 56, and its prevalence were 7.8, 7.8, 3.9, 2.0, 2.0, and 2.0%, respectively. The distribution of the most common Hr-HPV genotypes according to the cytological status is shown in Table 4. Whereas, the more prevalent of Lr-HPV infection or unknown oncogenic risk HPV genotypes among the respondents were HPV6, 11, 30, 34, 40, 42, 43, 54, 55, 61, 62, 64, 71, 74, 81, 84, 87, 89, 90, and 91. Only one beta-HPV was observed, HPV105, and four gamma-HPVs were observed including HPV101, 108, 147, and 121. Of those who were detected with gamma-HPVs, all had coinfection with alpha-HPVs; but no coinfection was found between beta-HPV and alpha-HPV in the present study subject. The distribution of mucosal HPV types (Hr-HPV and Lr-HPV) and cutaneous HPV types are shown in Table 3.

**Table 4:** Prevalence of the most common high-risk HPV genotypes by cytological abnormalities.

	**Cervical cytological status** ***N*** **(Col %)**					
		**LGSIL**		**HGSIL**		
**Hr-HPV (α)** **genotypes^#^**	**Normal** ***N*** **= 51**	**Inflammatory/ Atypia** ***N*** **= 164**	**CIN 1** ***N*** **= 27**	**CIN 2** ***N*** **= 29**	**CIN 3** ***N*** **= 29**	**Total** ***N*** **= 300**
HPV58	0 (0.0)	16 (9.8)	3 (11.1)	7 (24.1)	8 (27.6)	34 (11.3)
HPV52	4 (7.8)	14 (8.5)	1 (3.7)	8 (27.6)	6 (20.7)	33 (11.0)
HPV53	4 (7.8)	19 (11.6)	4 (14.8)	2 (6.9)	3 (10.3)	32 (10.7)
HPV16	2 (3.9)	8 (4.9)	4 (14.8)	2 (6.9)	11 (37.9)	27 (9.0)
HPV18	1 (2.0)	7 (4.3)	4 (14.8)	2 (6.9)	0 (0.0)	14 (4.7)
HPV51	0 (0.0)	7 (4.3)	2 (7.4)	4 (13.8)	0 (0.0)	13 (4.3)
All Hr-HPVs	9 (17.7)	69 (42.1)	13 (48.2)	25 (86.2)	25 (86.2)	141 (47.0)
HPV58 & others^∧^	2 (3.9)	49 (29.9)	5 (18.5)	13 (44.8)	11 (37.9)	80 (26.7)
HPV52 & others^∧^	6 (11.8)	47 (28.7)	3 (11.1)	15 (51.7)	8 (27.6)	79 (26.3)
HPV53 & others^∧^	6 (11.8)	48 (29.3)	5 (18.5)	8 (27.6)	6 (20.7)	73 (24.3)
HPV16 & others^∧^	4 (7.8)	40 (24.4)	6 (22.2)	9 (31.0)	14 (48.3)	73 (24.3)
HPV18 & others^∧^	3 (5.9)	40 (24.4)	6 (22.2)	9 (31.0)	4 (13.8)	62 (20.7)
HPV51 & others^∧^	2 (3.9)	41 (25.0)	3 (11.1)	11 (37.9)	4 (13.8)	61 (20.3)

# *High-risk HPV genotypes: included high-risk genotypes (16, 18, 31, 33, 35, 39, 45, 51, 52, 56, 58, and 59) and potential high-risk genotypes (53, 66, 67, 68, 70, 82)*.

∧ *Others: included high-risk genotypes (31, 33, 35, 39, 45, and 59) and potential high-risk genotypes (66, 67, 68, 70, 82)*.

#### Age-Specific Prevalence of HPV Infection in Cytological Abnormality

**Figure 2:**
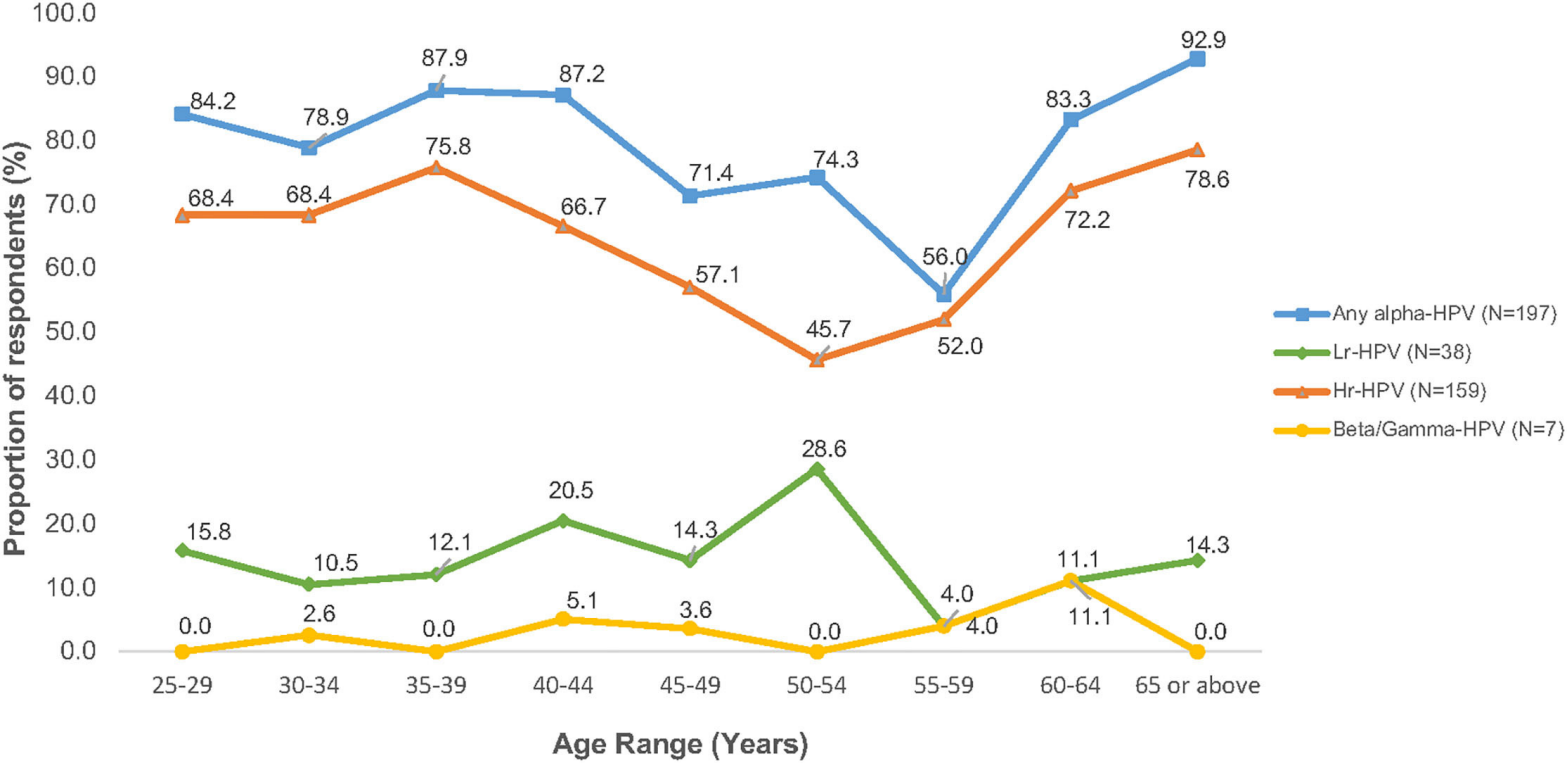
Age-specific prevalence of HPV infection among women with cytology abnormalities (*N* = 249).

Figure 2 shows that the age-specific prevalence of HPV (any-type HPV infection) among women with persistent abnormal cytology first peaked at age 35–39 years (87.9%), gradually declined to 56.0% at 55–59 years, with a second peak at age 65 or above (92.9%). A similar age distribution pattern was observed among Hr-HPV infection while low-risk HPV (Lr-HPV) infection revealed a rather distinct pattern, where the first and only peak occurred at 50–54 years (prevalence rate, 28.6%) and dropped abruptly at aged 55–59 years (prevalence rate, 4.0%). Of the 7 subjects with cutaneous HPV infection, all of them were also found among the women with persistent abnormal cytology. The age-specific beta/gamma-HPV prevalence was 2.6% at aged 30–34 years, 5.1% at aged 40–44 years, 3.6% at aged 45–49 years, 4.0% at aged 55–59 years, and 11.1% at aged 60–64 years.

#### Correlation Between HPV Infection and Cervical Cytological Status

Age-adjusted risk association analyses between cervical cytological status and HPV genotypes were performed. Significant associations were found between any-type HPV infection and abnormal cytology (OR = 3.7; 95% CI 2.0–7.1), and between Hr-HPV infection and abnormal cytology (OR = 6.3; 95% CI 3.0–13.5)–the details are shown in Table 5. However, when only Lr-HPV infection was taken into consideration, no statistically significant association was found with abnormal cytology (OR = 1.4; 95% CI 0.6–3.0). Similar association patterns were observed between LGSIL or HGSIL status and HPV genotypes. Due to the small-scale of cutaneous HPV detection, the causal relationship between different abnormal cytological statuses could not be further established in this study.

**Table 5:** Age-adjusted correlation between HPV infection and cervical cytological status.

	**Cervical cytological status**				
**HPV infection**	**Normal (*N* = 51) *N* (Col %)**	**Abnormal** **(*N* = 249)** ***N*** **(Col %)**	**OR**	**95% CI** ^∧^	***P*** **-value**
Negative	26 (51.0)	52 (20.9)	Ref		
Any HPV type	25 (49.0)	197 (79.1)	3.7	2.0–7.1	<0.001
Negative	26 (51.0)	52 (20.9)	Ref		
Low-risk HPV	13 (25.5)	38 (15.3)	1.4	0.6–3.0	0.436
High-risk HPV^#^	12 (23.5)	159 (63.9)	6.3	3.0–13.5	<0.001
	**Normal (*N* = 51) *N* (Col %)**	**LGSIL** **(*N* = 191)** *N* **(Col %)**	**OR**	**95% CI** ^∧^	***P*** **-value**
Negative	26 (51.0)	49 (25.7)	Ref		
Any HPV type	25 (49.0)	142 (74.4)	2.9	1.5–5.4	0.001
Negative	26 (51.0)	49 (25.7)	Ref		
Low-risk HPV	13 (25.5)	37 (19.4)	1.4	0.6–3.2	0.393
High-risk HPV^#^	12 (23.5)	105 (55.0)	4.4	2.1–9.6	<0.001
	**Normal (*N* = 51) *N* (Col %)**	**HGSIL** **(*N* = 58)** *N* **(Col %)**	**OR**	**95% CI** ^∧^	***P*** **-value**
Negative	26 (51.0)	3 (5.2)	Ref		
Any HPV type	25 (49.0)	55 (94.8)	19.0	5.1–71.3	<0.001
Negative	26 (51.0)	3 (5.2)	Ref		
Low-risk HPV	13 (25.5)	1 (1.7)	0.6	0.1–6.4	0.652
High-risk HPV^#^	12 (23.5)	54 (93.1)	45.9	10.7–196.0	<0.001

∧ *OR: odd ration; 95% CI: 95%*.

# *High-risk HPV infection: including high-risk genotypes (16, 18, 31, 33, 35, 39, 45, 51, 52, 56, 58, and 59), potential high-risk genotypes (53, 66, 67, 68, 70, and 82) and mixed HPV genotypes (high-risk, potential high-risk and low-risk) infection*.

## Discussion

The present study involved 249 women with cytological abnormalities with an overall prevalence of any-type HPV infection of 79.1%, while Hr-HPV infection was 63.9%, which is much higher than 7–10% of HPV infection in the general population (8, 9). The highly prevalent of HPV infection would be possibly due to the cytological abnormality status in our study subjects, which indicated that HPV-based screening as an estimated higher sensitivity, thus it could be adopted as an alternative to cytology testing (14). The study confirms that HPV infection is strongly associated with cervical precancerous lesions. In addition, it also provided additional information on cutaneous HPV infection to the development of cervical cancer. To date, a study on HPV prevalence among women with cytological abnormalities is scarce in the HK population, with only one study among Chinese women in South Coast China showed an overall prevalence of HPV at 42.3% (695/1,643) (10, 15). Thus, our findings not only provided a meaningful insight into the overview of molecular epidemiology of HPV infection among different abnormal cytological statuses in the precancerous stage of development but also explored the association between lifestyle behavior, cytological abnormality, and HPV infection.

This study shows that the prevalence of HPV infection (any-type and Hr-HPV) was significantly higher in younger aged women with cytological abnormalities, with an almost steady high HPV prevalence from age 25 years onwards until gradually declined at age 45 years. This might be explained that younger women were more likely to engage in health-risk behaviors, including cigarette smoking, abortion, sexual debut at an early age, and a greater number of lifetime sexual partners. While age-specific prevalence curve with two peaks of HPV infection was observed in many jurisdictions, including HK (9, 16, 17). However, their peaks of age-specific HPV infection were varied. The age-specific HPV prevalence across other Provinces of China showed a “U-shaped” pattern with the first peak detected at 19 years or below and declined at 30 years until reached a second peak at 60 years or older (17). In the contrary, despite that Hong Kong is a city and a special administrative region of China, our previous study revealed a distinct “Bimodal” pattern with two peaks of HPV infection were detected at 26–30 years and 46–50 years in general population, respectively (14). A similar bimodal pattern was also observed in this study with two major peaks of HPV infection among women with abnormal cytology at aged 35–39 years, and an accelerated risk of both any-type and Hr-HPV infection at age 65 or above. This bimodal pattern implies a 10-year shifting of HPV prevalence between women in the general population and those with cytological abnormalities (14) which provided a good support for applying HPV testing as primary cervical cancer screening for early detection of cancer development. In addition, the findings also suggest that cervical cancer screening for women aged 65 or above could be recommended. However, at present guidelines of the government programme on cervical cancer screening in HK are targeted at age 25 or the time of commencing sexual activity until the age of 64 (18).

The most commonly detected HPV genotypes were HPV58 the most prevalent, followed by 52, 53, 16, 51, and 18 among the respondents in the present study. Hence, an updated epidemiology profile of the age-specific HPV prevalence and variation of type-specific HPV distribution was supplemented to our previous similar studies conducted in the last decade (10, 16). In terms of type-specific HPV distribution, similar results were found in a few retrospective studies where the most prevalent Hr-HPV type was HPV52, followed by HPV16 and HPV58 (19), and other Asia countries such as Singapore (20), Thailand (21), and South Korea (22). The consistent type-specific HPV prevalence in the China study (18) was expected, as about 92.0% of the population were ethnic Chinese in HK (23). However, a different type-specific group was found when compared to Western countries, where the most prevalent HPV oncogenic types were HPV16, followed by HPV18, 45, 33, and 31 among women with cervical lesions in the United Kingdom, United States of America, and Turkey (24, 25). Interestingly, considerable proportions of Hr-HPV51 and potentially Hr-HPV53 were observed in women with cervical cytological abnormalities in this study in which these two HPV genotypes were not prevalent in either other Asian nor Western countries. However, both HPV51 and HPV53 are not covered by any of the three currently registered HPV vaccines (2-valent vaccine: HPV16 and 18; 4-valent vaccine: HPV6, 11, 16, and 18; and 9-valent vaccine: HPV6, 11, 16, 18, 31, 33, 45, 52, and 58) in HK (26). Thus, further investigation on evaluating the efficacy of the currently registered HPV vaccines in HK is needed.

The present study also revealed that any-type HPV or Hr-HPV infection was significantly associated with cytological abnormalities (*p* ≤ 0.05). This is in line with previous studies that cervical HPV acquisition is linked to the development of HGSIL precursor lesions; hence, subsequently, progress to cervical cancer (27). Worldwide, oncogenic HPV16, 18 and HPV6, 11, 16, 18, 31, 33, 45, 52, and 58 were associated with approximately 70 and 90% invasive cervical cancer, respectively (28). We have therefore further evaluated the association between different cytological statuses and specific HPV genotypes, especially the most prevalent HPV types in HK. This study showed that the most common Hr-HPV types among women with HGSIL (CIN2 and CIN3) were HPV58 and 52, while among those with LGSIL (inflammatory/atypia and CIN1) HPV53, 16, and 18 with distinct oncogenic HPV types were identified in HK.

Our observation of cutaneous HPV types (Beta-β and Gamma-γ) at mucosal sites is among the first studies to do so conducted in HK. We identified all respondents who were detected with cutaneous HPV infection also had cytological abnormalities. Thus, it confirmed the existence of HPV coinfection between mucosal HPV and cutaneous HPV genotypes in cervical cytological abnormalities. Despite a limited number of observations were found and only five HPV genotypes were identified, namely, HPV105 (beta-1 type), HPV101, 108, 147, and 121 (gamma-6, 8, and 10 types). This provides a unique and additional insights into the study of natural history and clinical picture of cutaneous HPV infection in HK. However, due to the small-scale of samples' detection, a causal relationship between different abnormal cytological statuses could not be established in the present study, and further studies with larger sample sizes are needed to draw a confirmative conclusion.

Cutaneous beta-HPV types are considered to be fairly similar to Hr-HPV types, as they both attribute to the development of carcinogenesis (17, 29). Of which beta-1 and beta-2 species are the most prevalent subdivided HPV types that infect the skin; hence, increasing the risk of developing skin lesions or even malignancies. However, little is known about the role of this cutaneous HPV in the genital mucosa. In a previous study on the detection of HPV genotypes using samples in cervical swabs, results suggest that beta-PVs and gamma-PVs could be possible carcinogenic cofactors in cervical infections (5). However, further investigation to determine the significance of HPV coinfections is needed. Our study thus highlights the importance of conducting future research on the relationship between HPV genotypes including cutaneous HPV types and squamous cell carcinoma (SCC) to the development of cervical cancer (CC) preventive strategies among Chinese populations in South-East Asia.

In HK, an updated version of the guidelines for cervical cancer prevention and screening was recommended to physicians in public hospitals in November 2016. The new revision provided guidance on the use of HPV testing either as a stand-alone test or as part of “co-test” with cytology for primary screening (18), and in addition on the feasibility of HPV self-sampling (14) for women aged 30 years or above. This study suggests that the bimodal pattern of age-specific HPV prevalence had a 10-year shifting between women in the general population and those with cytological abnormalities. Thus, it implies that applying HPV testing as primary cervical cancer screening for early detection of cancer development is a sound strategy for the way forward for Chinese population. In recent years, the introduction of HPV testing for high-risk oncogenic HPV types as primary screening or co-testing in the national cervical cancer screening program has been endorsed in many countries, such as the United Kingdom (30), Australia (31), the United States (32), and within the European Union including Denmark, Finland, Italy, Sweden, Romania, and Portugal (24). Several studies suggested that HPV testing generally has greater sensitivity in detecting precancerous lesions than cytology results (33). The World Health Organisation (WHO) also recommends HPV detection tests as an alternative for cervical cancer screening (34). Furthermore, the Centre for Health Protection of the Department of Health announced that starting from the 2019/20 school year, eligible female primary school students of suitable ages would be provided with the HPV vaccine under the Childhood Immunization Programme (HKCIP) for cervical cancer prevention (35). However, there is a potential shift in prevalence of the HPV types among the local population which may affect the efficacy of the current national immunization/vaccination programme (36). Thus, it is a good time to develop a databank for recording the population in terms of their HPV infection, its genotypes, and the cytological status for monitoring the molecular epidemiology of HPV infection and cervical cancer in HK. This study provides a constructive information on the development and applicability of HPV-based screening strategies in the HK population.

## Conclusion

This study provided an updated epidemiologic information on HPV infection among women with cytological abnormalities and demonstrated that specific HPV genotypes were associated with various stages of cervical abnormalities. It also confirmed the presence of cutaneous HPV types (Beta-β and Gamma-γ) in cervical infections. Our research also revealed that there was an association between age and HPV infection, with a significantly higher prevalence of HPV infection (any-type and Hr-HPV) among younger women. In terms of the type-specific HPV distribution in HK, our analysis revealed a similar pattern as in other Asian countries but varies in Western countries. Our research, therefore, promotes insights into the development of cervical cancer prevention strategies and the applicability of HPV-based screening programme in HK.

## Data Availability Statement

The datasets are available from the corresponding author on reasonable request. The data are not publicly available due to their containing information that could compromise the privacy of research participant.

## Ethics Statement

The studies involving human participants were reviewed and approved by the Joint Chinese University of Hong Kong–New Territories East Cluster Clinical Research Ethics Committee (The Joint CUHK-NTEC CREC). The patients/participants provided their written informed consent to participate in this study.

## Author Contributions

EW and PC: conceptualization and methodology. AC, AW, and PY: formal analysis. EW, PC, AC, and AW: investigation and writing—reviewing and editing. EW, AC, PY, and AW: data curation. EW, AW, and AC: writing—original draft preparation. AC and AW: project administration. AY, ZC, and PC: laboratory analysis. All authors have read and agreed to the published version of the manuscript.

## Funding

This study was funded by the Hong Kong Food and Health Bureau through Commissioned Programmes on Control of Infectious Diseases (Phase III) (Reference No. CU-15-C7). The financial support of the Centre for Health Systems and Policy Research is from the Tung's Foundation.

## Conflict of Interest

The authors declare that the research was conducted in the absence of any commercial or financial relationships that could be construed as a potential conflict of interest.

## Publisher's Note

All claims expressed in this article are solely those of the authors and do not necessarily represent those of their affiliated organizations, or those of the publisher, the editors and the reviewers. Any product that may be evaluated in this article, or claim that may be made by its manufacturer, is not guaranteed or endorsed by the publisher.
